# Chronic disease management: policy design based on service design methods

**DOI:** 10.3389/fpubh.2025.1704995

**Published:** 2026-01-13

**Authors:** Jie Xue, Wei Jiang, Xiaoyu Xu, Han Tan, Guosheng Wang

**Affiliations:** 1 Academy of Arts & Design, Tsinghua University, Beijing, China; 2 National Centre for Non-Communicable Disease Control and Prevention, Chinese Centre for Disease Control and Prevention, Beijing, China

**Keywords:** chronic disease, diabetes mellitus, health policy, hypertension, mixed methods, patient-centered care, primary health care

## Abstract

**Introduction:**

Chronic diseases, such as hypertension and diabetes, impose significant burdens on health systems and communities.

**Methods:**

To inform implementation-oriented public health strategies, this study utilized a mixed-methods design that integrates population-level administrative data, patient-reported experiences, and qualitative inquiry in Xiling District, Yichang, China. The data sources included 93,146 adults with hypertension and 32,050 adults with diabetes from the district’s chronic disease management information system, a survey of 518 patients, and qualitative data from semi-structured interviews (*n* = 10) and focus group discussions (*n* = 19). The analytic approach employed descriptive statistics to construct and assess care cascades for diagnosis, enrollment, standardized management, treatment, and clinical control, alongside reflexive thematic analysis of qualitative data, with findings synthesized through a service-design lens.

**Results:**

The findings indicated (1) significant drop-offs along care cascades, particularly in enrollment in basic public health services and standardized management; (2) user-journey evidence suggesting limited process visibility, low perceived value of follow-up services, and fragmented information flow, which elucidate the observed cascade gaps; and (3) stakeholder feedback highlighting inadequate coordination among service providers.

**Discussion:**

By synthesizing quantitative and qualitative insights, the study proposes a three-layer service-design framework—users, service processes, and service provision—to guide the redesign of chronic disease management services. These findings illustrate how the integration of administrative data with user experience insights can lead to more actionable and user-centered policy design.

## 1 Introduction

In recent decades, China’s rapid socioeconomic development has substantially improved living standards. However, accelerated urbanization and population aging have contributed to a rising burden of chronic non-communicable diseases (NCDs), particularly hypertension and diabetes, which have been identified as major contributors to morbidity and mortality in China and globally (1–3). According to the 2018 China Chronic Disease and Risk Factor Surveillance Report, the prevalence of diabetes among adults aged 18 and older in China was 11.9%, with awareness, treatment, and control rates at 38.0, 34.1, and 33.1%, respectively (4). Currently, China is home to over 200 million individuals with hypertension and more than 100 million with diabetes. Concurrently, the proportion of deaths attributed to chronic diseases among total deaths nationwide continues to rise.

Chronic diseases, characterized by long-term or progressive declines in organ function, primarily affect middle-aged and older populations (5). Their occurrence is closely linked to biological, psychological, and social environmental factors, as well as individual lifestyles (6). High-risk factors arise from personal behaviors, lifestyle choices, family conditions, societal conditions and so on (7–10). Chronic diseases such as hypertension and diabetes are characterized by prolonged duration and substantial consumption of societal resources. Due to their high incidence, low awareness, and poor control rates (11), these conditions have emerged as critical public health challenges in China, posing significant threats to population health.

Effectively preventing and controlling chronic diseases, while formulating context-specific policies, is a key focus of current research (12). The World Health Organization has proposed a six-pillar theoretical framework for health systems (13), which encompasses governance structures, health financing, health workforce, health information systems, medicines and technologies, and health service delivery models. This framework aids countries in identifying gaps in their chronic disease management strategies. However, the challenges associated with chronic disease management are complex and diverse, constrained by the specific conditions of different countries and regions. Traditional quantitative and qualitative analysis methods address only superficial issues, failing to systematically examine the underlying mechanisms of chronic disease services across various regions. This limitation hinders comprehensive and effective problem analysis, thereby impacting the precision of intervention policy formulation. Recently, Zhao et al. constructed a performance evaluation index system for the management of chronic diseases based on medical and preventive integration, which can act as a useful tool for diversified subjects to find the loopholes and weak points in chronic disease management (14). Besides, the influence of patient self-efficacy on value co-creation behavior and outcomes in chronic disease management could not neglected (15). Similar limitations of predominantly top-down policy approaches have been reported in public health and governance research (16, 17).

Service Design is a user-centered, systematic design approach aimed at creating efficient, useful, and satisfying services by optimizing service processes, resources, touchpoints (the tangible and intangible interfaces through which users engage with a service. These touchpoints encompass physical environments, digital platforms, communication channels, and human interactions. Collectively, they shape the user’s journey and emotional response to the service), and user experiences. It emphasizes the holistic nature of services, encompassing both visible aspects (such as interfaces and environments) and invisible components (such as backend processes and employee collaboration), thereby ensuring value for both users and providers (18). As an emerging discipline, Service Design has rapidly evolved in developed countries since the 1990s, becoming one of the key design directions for addressing socio-economic transformation (19). It extends beyond traditional commercial services into public service domains, particularly in healthcare (20). With the growing global influence of Service Design, an increasing number of countries and regions are exploring its integration with healthcare policy design. Recent reviews and policy-oriented studies have documented the institutionalization of service design within health systems and public-sector innovation frameworks (21).

In addressing healthcare challenges, Service Design thinking effectively visualizes and concretizes problems, thereby defining their core aspects to enhance the rationality and feasibility of policy design. It has been reported that the innovative mindsets and methodologies proposed by Service Design can influence key stakeholders within the healthcare industry, prompting them to reconsider and optimize medical service policies through interdisciplinary collaboration (22, 23). The core of policy design focuses on addressing conflicts and issues of interest. Conflicts primarily manifest as tensions between supply and demand, while interests pertain to the long-term and immediate benefits for the general public. For instance, in the formulation of chronic disease policies, the state aims to reduce the incidence of chronic diseases in China through targeted policy interventions. This approach mitigates the economic losses associated with such conditions, thereby contributing to China’s economic and social development.

**Figure 1:**
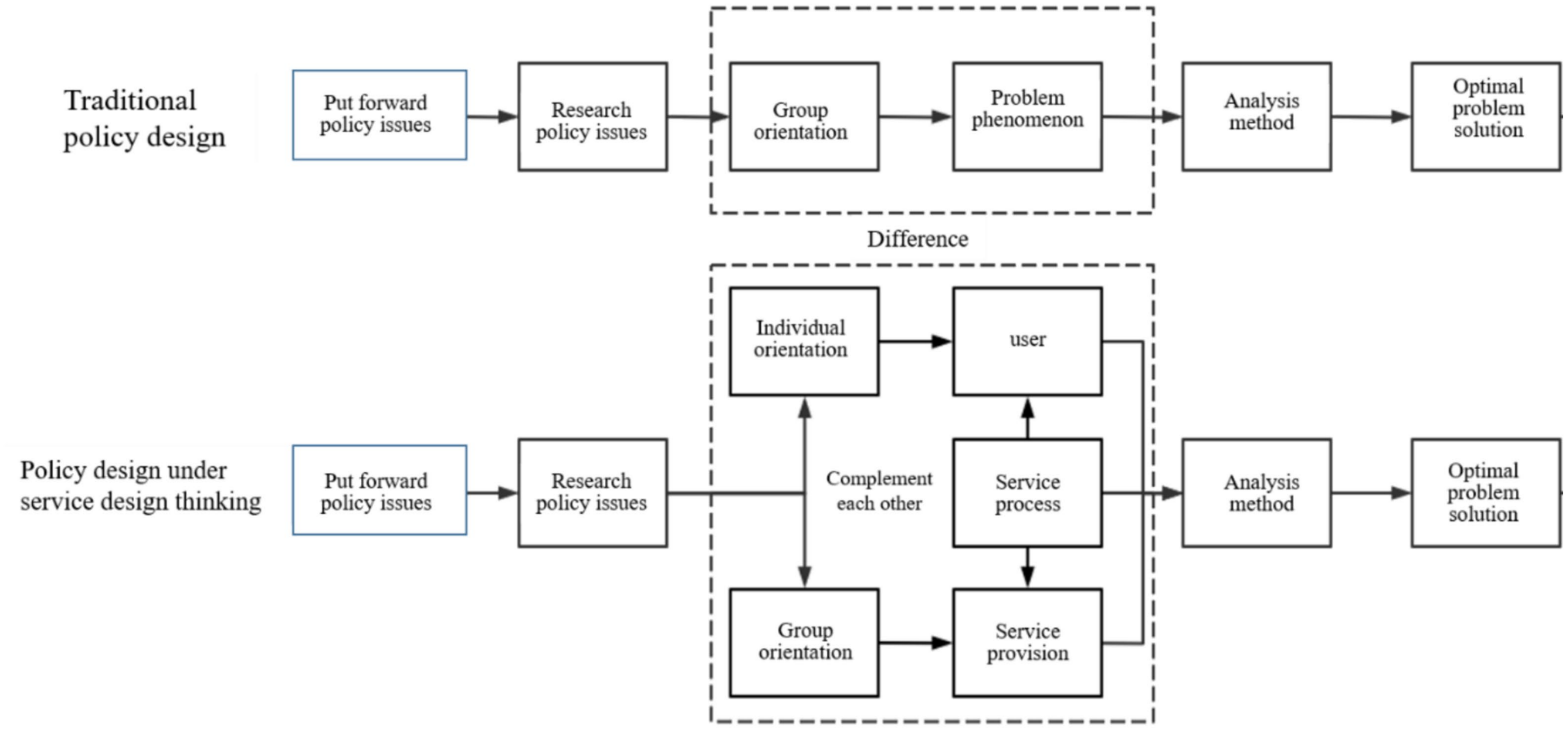
Comparison of traditional policy design and service-design–informed policy design: analytical focus and process logic.

Figure 1 illustrates the conceptual differences between traditional policy design and policy design informed by service design principles. Traditional policy design is characterized by a predominantly top-down and group-oriented analytical sequence that focuses on problem identification, analysis, and policy formulation. In contrast, the service-design-informed approach integrates user-centered perspectives, service-process analysis, and iterative feedback, emphasizing the interconnected roles of users, service processes, and service provision. The figure provides a schematic overview of how service design complements, rather than replaces, conventional policy design by operationalizing participatory and implementation-oriented principles.

The traditional policy design process consists of four main stages: problem identification, research, analysis, and policy formulation, as illustrated in Figure 1. Traditional policy design methods primarily emphasize group orientation by examining common issues that arise within groups and focusing on surface-level phenomena. The identification and determination of policy issues are carried out through three methods. The approach is qualitative, which involves subjective analysis or expert judgment to summarize and organize the manifestations of problems, thereby formulating corresponding solutions. The second approach is quantitative, employing quantitative research to investigate issues, using data to reflect specific problems and addressing them sequentially based on their quantified severity. The third approach combines qualitative and quantitative methods, offering a comprehensive and accurate reflection of both subjective and objective issues. In existing policy research processes, this combined method is typically utilized for issue analysis and measure formulation. The policy design approach based on service design operates concurrently with the traditional policy design approach, encompassing four main components: problem identification, problem research, problem analysis, and solution measures, as illustrated in Figure 1. In contrast to traditional policy design, this approach integrates service design thinking and methodologies. Guided by service design principles, this paper argues that policy design should balance collective and individual needs, emphasizing the often-overlooked individual-oriented perspective in traditional policy design. The analysis and framework for chronic disease policy design are structured around the three core elements of service design: users, service processes, and service delivery, as shown in Figure 1.

Policy design has often been portrayed as a linear, top-down sequence of problem identification, analysis, and formulation (24). However, in modern public governance—particularly in the health sector—policy development increasingly adopts participatory and iterative logics. Frameworks such as the Theory of Change (ToC), co-production models, and pilot-based social experiments illustrate how contemporary policy design integrates bottom-up stakeholder engagement and adaptive learning cycles (25). Accordingly, this study does not frame service design as a replacement for traditional policy design but as an evolutionary complement that operationalizes participatory principles through user-centered visualization, co-creation, and feedback loops.

Policy design emphasizes the development of policy instruments, governance arrangements, and implementation strategies, while service design focuses on user-centered operational processes. Consequently, service design can be interpreted as an operational methodology that supports and enhances policy implementation. In this context, service design offers practical tools—such as visualization, co-creation, iterative prototyping, and context-based user insights—that can strengthen the delivery and adaptability of policy instruments within complex health systems (26, 27). Internationally, institutions such as the OECD Observatory of Public Sector Innovation (OPSI) exemplify the institutionalization of service design within policy innovation frameworks (21). These examples illustrate how integrating service design into health governance allows governments to refine policies through iterative learning, user testing, and evidence-based adaptation (28–30). This conceptual linkage underpins the present study’s integration of service design principles into chronic disease management reform. This framework conceptualizes service design as an implementation-oriented methodological system. Unlike traditional policy design models, which emphasize group-level problem diagnosis and follow a predominantly linear analytical sequence, this framework systematically integrates user insights, service-process analysis, and iterative co-creation into the core of policy research and implementation. By embedding these operational tools within the policy design process, the framework enhances the adaptability, practicality, and user-centered policy implementation, offering a more evidence-based and experience-driven approach to improving policy performance in complex health systems (31, 32).

We integrate population-level records to identify gaps in the cascade, patient-reported experiences to comprehend the perceptions underlying these gaps, and qualitative inquiries to uncover the mechanisms of the processes. These findings are then translated into service-design artifacts that inform policy actions. The research design integrates quantitative data analysis with qualitative insights derived from patient interactions, aiming to construct a comprehensive framework that supports evidence-based service innovation. The study is conducted as follows: (1) The quantitative data on patient demographics, disease characteristics, and service utilization are collected and analyzed using descriptive and statistical methods to identify key trends and challenges in chronic disease management. (2) The qualitative methods—including in-depth interviews and focus group discussions (FGDs)—are employed to capture patients’ emotional and behavioral responses across different stages of their care journey. These qualitative data are analyzed using thematic analysis to extract critical pain points and service gaps. (3) The findings from both the quantitative and qualitative analyses are synthesized. This integrative analysis enables the visualization of patient pathways and the identification of opportunities for system-level service improvement. Overall, the study design emphasizes a participatory and iterative process, combining empirical evidence with the principles of design thinking to generate actionable insights for enhancing chronic disease care delivery.

## 2 Methods

This mixed-methods study was conducted in the Xiling District of Yichang City, Hubei Province, China. It integrated administrative health records with patient surveys, in-depth interviews, and FGDs. The primary aim of the study was to identify gaps in the care cascade for hypertension and diabetes management, as well as to explore the underlying experiential and process-level mechanisms using service design methods.

### 2.1 Administrative and clinical data

The administrative datasets were sourced from the chronic disease management information system of the Yichang Health Bureau. Key variables included patient demographics (age, sex), diagnosis records (ICD-10 codes), clinical measurements (blood pressure, fasting glucose), treatment records, medication prescriptions, follow-up records, and participation in basic public health services. Data cleaning involved the removal of duplicates, logical consistency checks, exclusion of records with missing identification information, and the elimination of biologically implausible values (e.g., systolic blood pressure ≥260 mmHg; fasting glucose ≥30 mmol/L). Only adults aged 35 years and older with confirmed hypertension or diabetes were included in the analysis. Records lacking diagnosis dates or complete follow-up information were excluded from cascade analyses.

Prior to researcher access, all direct identifiers (such as names, ID numbers, phone numbers, and addresses) were removed. Only de-identified analytic datasets were utilized. The data covered the period from January to December 2022. Given that this timeframe coincided with China’s later phases of COVID-19 control and reopening, service utilization patterns may have been affected by clinic closures, postponed follow-ups, or disrupted access to medications. Potential distortions related to COVID-19 were taken into account when interpreting the gaps in the cascade.

Hypertension was defined according to the Chinese Guidelines for Hypertension Management, which stipulate that systolic blood pressure (SBP) must be ≥140 mmHg, diastolic blood pressure (DBP) must be ≥90 mmHg, or the individual must be currently using antihypertensive medication (33). A patient was classified as having diabetes if one or more of the following criteria were met: ICD-10 diagnosis codes E10–E14; laboratory criteria indicating fasting plasma glucose (FPG) ≥ 7.0 mmol/L or HbA1c ≥ 6.5%; and chronic antidiabetic medication use, excluding likely non-diabetes cases (such as metformin for polycystic ovary syndrome/prediabetes, GLP-1 receptor agonists for obesity, and SGLT2 inhibitors for heart failure/chronic kidney disease) (34). Standardized management adheres to the National Basic Public Health Service Standards (Third Edition), which mandate at least four follow-up visits per year, including documented health education, medication reviews, and risk assessments (35). Cascade percentages were computed sequentially at each stage: diagnosis, service enrollment, standardized management, treatment, and control.

### 2.2 Survey participants and sampling

#### 2.2.1 Sampling strategy

This study employed purposive sampling to recruit chronic disease patients exhibiting diverse characteristics, including variations in age groups, sex, disease types, and management status. Participants were recruited from community health centers and hospitals located in Xiling District between November 2021 and February 2023.

##### 2.2.1.1 Eligibility criteria

Inclusion Criteria: (1) Age ≥ 35 years. (2) Clinically diagnosed with hypertension or diabetes in a stable or recovery phase. (3) Mentally alert, possessing adequate comprehension and communication abilities. (4) Willing to participate voluntarily and have provided written informed consent.

Exclusion Criteria: (1) Presence of diagnosed cognitive impairment or psychiatric disorders. (2) Critically ill patients who are unable to cooperate with the questionnaire procedures.

#### 2.2.2 Sample size and ethical approval

This study employs a descriptive cross-sectional design for its survey component. The sample size was determined based on feasibility, expected response variability, and the necessity for stable descriptive estimates within the target population. The study received approval from the Medical Ethics Committees of the Chinese Centre for Disease Control and Prevention, the Guangdong Provincial CDC, and the Second Affiliated Hospital of Harbin Medical University (Approval No. KY2021-319).

### 2.3 Survey procedures

This questionnaire was self-developed based on a comprehensive literature review and expert consultation. The questionnaire comprised five sections. The first section collected basic demographic information, including gender, age, education, living situation, and type of medical insurance. The second section focused on disease and treatment status, such as type of chronic disease, time since diagnosis, treatment methods, care-seeking behavior, and financial burden. The third section assessed daily self-management behaviors, including monitoring indicators, sources of health information, diet control, and physical activity. The fourth section examined utilization and perceptions of primary health services, covering health records, free check-ups, family doctor services, follow-up care, and participation in community health activities. The fifth section investigated patient needs and satisfaction, focusing on service satisfaction, preferred health services, and confidence in disease self-management.

Before distributing the questionnaires, we contacted the nursing departments of all participating hospitals to obtain approval and support from their administrators. The investigators then visited clinical departments and used standardized instructions to explain the research purpose and the meaning of survey to each eligible adult patient with chronic diseases at their bedside. After obtaining written informed consent, they administered the questionnaires face-to-face and collected them immediately. The investigators verified the completeness of each questionnaire on-site and prompted participants to complete any missing items to ensure data quality. For patients who had difficulty in completing the questionnaire independently, the investigators recorded their responses verbatim based on oral input. After excluding those with patterned responses or logical inconsistencies, 518 valid questionnaires were ultimately collected.

### 2.4 Quantitative analyses

Quantitative data were analyzed using SPSS Statistics version 26.0. Descriptive statistics were generated for all variables, including frequencies and percentages for categorical variables (e.g., gender, disease type) and means with standard deviations for continuous variables. Administrative data related to hypertension and diabetes cascades were summarized using cross-tabulations. Quantitative analysis served a formative diagnostic function to identify cascade gaps and to guide qualitative and service-design inquiry; no causal inference was intended.

### 2.5 Qualitative component (interviews and FGDs)

A total of 10 in-depth interview participants (hypertension and/or diabetes) and 19 FGD participants (primary-care staff, administrators, community leaders, patient representatives) were recruited through community health centers and hospitals using purposive sampling to maximize diversity of experiences. The sociodemographic and clinical characteristics of interview participants are summarized in Supplementary Table S1.

Interviews and focus group discussions (FGDs) were conducted by two researchers trained in public health and qualitative inquiry. Their lack of clinical duties within the local health system mitigated potential power-related biases. Data were collected through in-person interviews held at community health centers or private meeting rooms between 2021 and 2023. Each interview lasted between 30 and 60 min, while each FGD lasted from 60 to 90 min. Semi-structured interview and FGD guides were utilized (see Supplementary material), encompassing topics such as the diagnostic journey, self-management practices, service utilization, medication access, interactions with primary care providers, barriers and facilitators, emotional responses, and expectations for care. All sessions were audio-recorded with participants’ consent, transcribed verbatim, and anonymized for analysis.

### 2.6 Qualitative analysis

A reflexive thematic analysis framework guided the qualitative analysis. Two coders independently performed line-by-line open coding of all transcripts, followed by axial coding to cluster related codes into subthemes aligned with service-design logic, and selective coding to develop higher-order themes that informed the construction of user personas and journey map interpretations. Qualitative data were managed using Excel and manually cross-checked to ensure analytic traceability. Any coding discrepancies were resolved through discussion and consensus, with a senior researcher providing adjudication when necessary. The team determined that thematic saturation was achieved when no new codes emerged across two consecutive interviews and focus group discussions, as documented in the saturation memo provided in the Supplementary material. Reporting of the qualitative and mixed-methods components adhered to established standards, including COREQ for interviews and focus groups, SRQR for qualitative research, and GRAMMS for mixed-methods integration (36–38).

**Figure 2:**
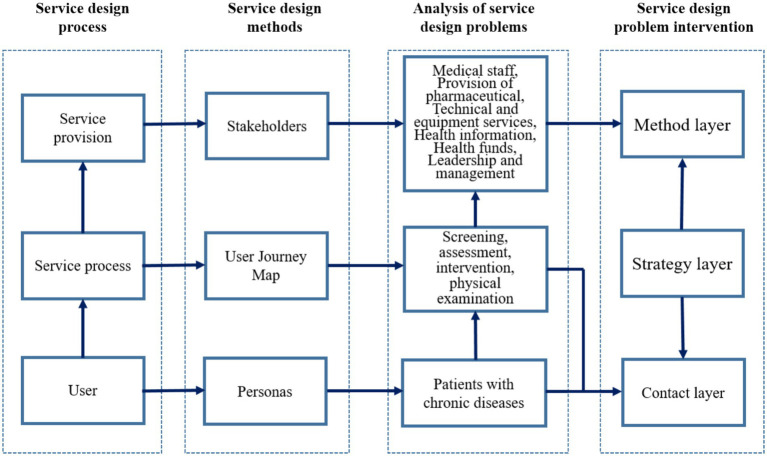
Logic diagram of chronic disease policy research from a service design perspective: integration of data sources and analytical layers.

Figure 2 presents the overall analytical framework of the study from a service design perspective. The diagram illustrates the integration of population-level administrative data, patient survey data, and qualitative insights from interviews and focus group discussions (FGDs) to inform various service design tools, including stakeholder analysis, user personas, and user journey mapping. Collectively, these tools support the identification of experiential and process-level gaps in chronic disease management, guiding the formulation of strategic principles, operational actions, and service touchpoints.

## 3 Results

### 3.1 Administrative dataset findings

**Figure 3:**
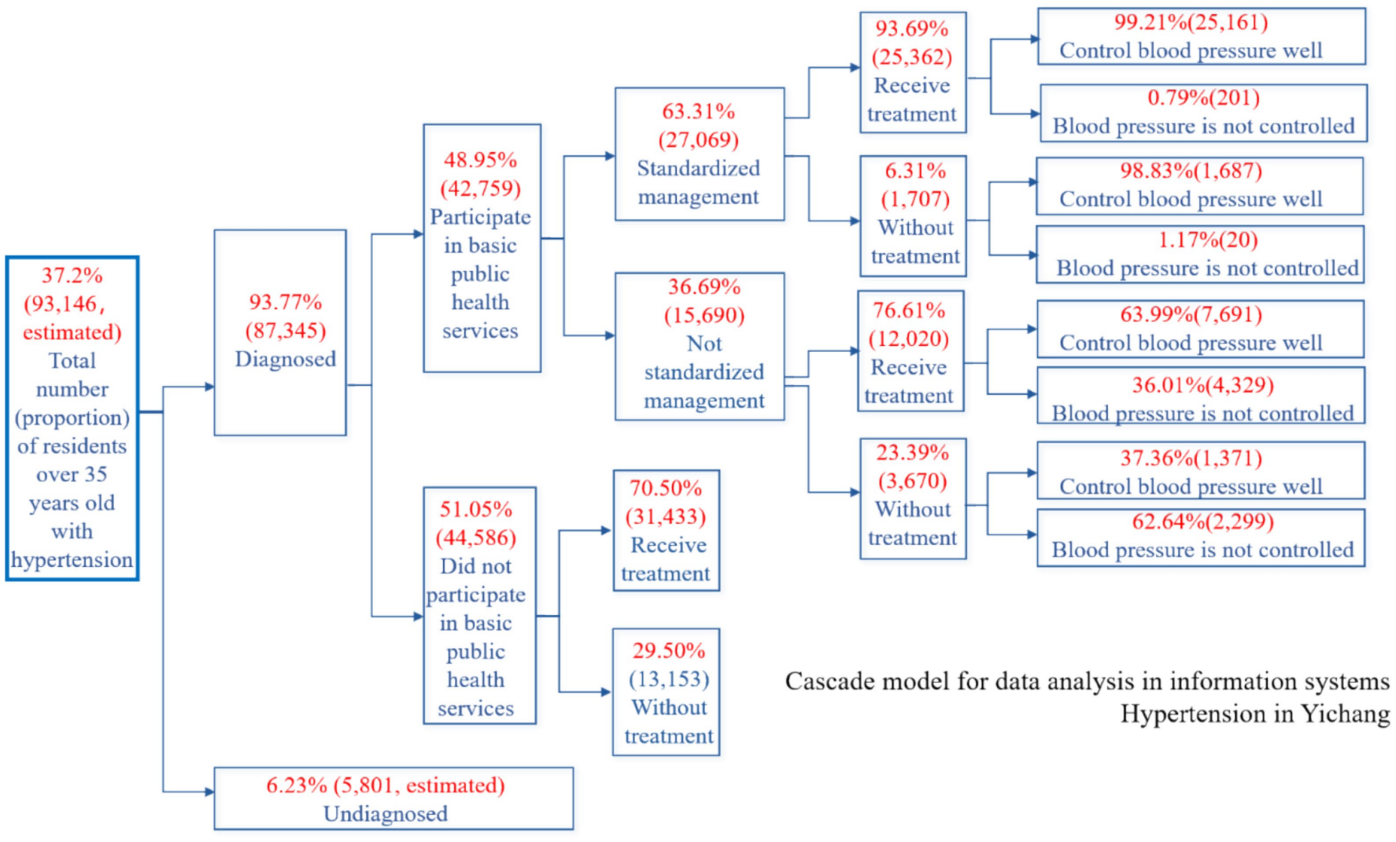
Hypertension care cascade—Xiling District adults aged ≥35 years, 2022: Enrollment and standardized-management gaps.

Figure 3 illustrates the hypertension care cascade for adults aged 35 years and older in Xiling District, Yichang City, China, in 2022. This cascade delineates the sequential stages of hypertension management, which include the identification of hypertension, recorded diagnosis, enrollment in basic public health services, standardized management, treatment, and blood pressure control.

Data were obtained from the Chronic Disease Management Information System of the Yichang Health Bureau. Percentages at each stage are calculated using the preceding stage as the denominator. ‘N’ indicates the number of individuals included at each stage. Hypertension Cascade.

Among adults aged ≥35 years (Figure 3), 37.2% met the criteria for hypertension. Of those diagnosed with hypertension, 93.77% had a recorded diagnosis. Furthermore, 48.95% of these diagnosed patients were enrolled in basic public health services. Among the enrollees, 63.31% met the criteria for standardized management. Treatment coverage was 93.69% among patients receiving standardized management, compared to 76.61% among those who were not. Blood pressure control was achieved in 99.21% of patients under standardized management, while only 63.99% of non-standardized patients achieved similar control. These differences reflect patterns observed within the administrative data structure; however, no causal inferences can be drawn.

**Figure 4:**
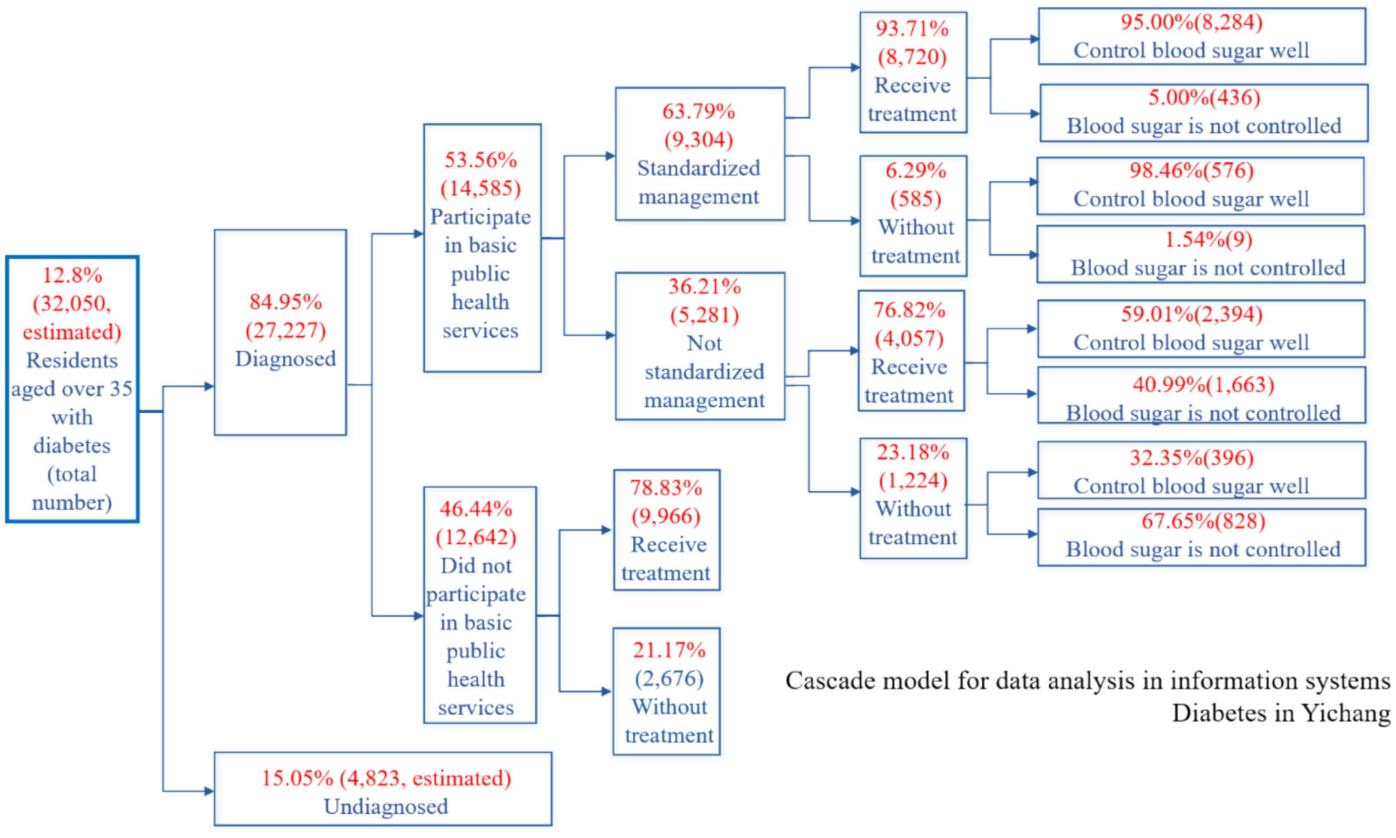
Diabetes care cascade—Xiling District adults ≥35 y, 2022: Enrollment and standardized-management gaps.

Figure 4 presents the diabetes care cascade among adults aged ≥35 years in Xiling District, Yichang City, China, in 2022. The cascade illustrates sequential stages including identification of diabetes, recorded diagnosis, enrollment in basic public health services, standardized management, treatment, and blood glucose control.

Data were derived from the chronic disease management information system of the Yichang Health Bureau. Percentages at each stage are calculated using the preceding stage as the denominator. N indicates the number of individuals included at each stage.

#### 3.1.1 Diabetes Cascade

Among adults aged 35 years and older (Figure 4), 12.8% met the criteria for diabetes, while 84.95% had a recorded diagnosis. Of those diagnosed, 53.56% were enrolled in public health services, and 63.79% of enrollees were managed according to standardized protocols. The treatment coverage was 93.71% for patients under standardized management and 76.82% for those under non-standardized management. Blood glucose control was achieved in 95.00% of patients managed under standardized protocols compared to 59.01% of those under non-standardized management. It is important to note that these differences reflect documented cascade stages and do not imply causality.

### 3.2 Survey findings

Among 518 surveyed chronic disease patients: The sample contained a range of ages, disease durations, and clinical conditions (see Methods). “Self-management” in this study refers specifically to medication-taking routines, diet, physical activity, and routine monitoring (as assessed in the survey tool).

Examples of survey-derived descriptive findings include: 40% reported high-salt dietary habits. 32% reported difficulties remembering medication instructions. 35% expressed uncertainty about where to obtain follow-up services. These values serve as contextual descriptors and are not used to infer behavioral determinants.

### 3.3 Qualitative findings

This paper will apply service design methods to conduct an in-depth investigation into chronic disease-related issues, adopting a user-centered approach through tools and methods such as user persona development, user journey mapping, and stakeholder analysis. The goal is to propose an integrated chronic disease management intervention and solution that optimizes user experience, policy implementation efficiency, and overall value maximization.

#### 3.3.1 User-centric stakeholder analysis

User-centered stakeholder analysis is a fundamental methodological approach in the field of service design. This method systematically identifies and examines stakeholders who are directly or indirectly involved in the service ecosystem, focusing on their needs, expectations, and degrees of influence. Through this analytical process, designers can achieve a more balanced consideration of diverse stakeholder interests during service planning and decision-making. Furthermore, by emphasizing the centrality of users in the design process, user-centered stakeholder analysis facilitates a holistic understanding of stakeholder interrelationships, enhances overall service experiences, fosters value co-creation, and provides a robust theoretical foundation for subsequent service process optimization and strategic development. To gather broader consensus and policy recommendations, the research team organized 19 FGDs involving primary healthcare institution administrators, public health experts, community leaders, and patient representatives. These discussions centered on key issues such as the accessibility, continuity, and equity of chronic disease management services. Through collaborative workshop approaches, we conducted preliminary stakeholder mapping. The objectives of the stakeholder analysis are as follows: (1) to introduce service design thinking and guide participatory design; (2) to identify stakeholders related to basic public health services, thereby enabling a better understanding of their current state. We mapped four key areas of chronic disease management within basic public health services: health promotion, medical care, pharmaceuticals, and health insurance. By placing patients at the center of the stakeholder framework, we categorized stakeholders based on their proximity to patients into three groups: direct service providers (including doctors, nurses, hospitals, pharmacies, online pharmaceutical platforms, media, family members, health insurance bureau staff, hospital reimbursement clerks, retail pharmacies, etc.), indirect service providers (such as neighborhood committees, local disease control centers, health bureaus, township hospitals, community health centers, pharmaceutical R&D institutions, hospital decision-makers, medical suppliers, drug logistics providers, health insurance settlement systems, commercial insurance companies, NGOs, etc.) and support service providers (including health insurance bureaus, disease control centers, HR departments, medical schools, education departments, and pharmaceutical regulatory agencies).

**Figure 5:**
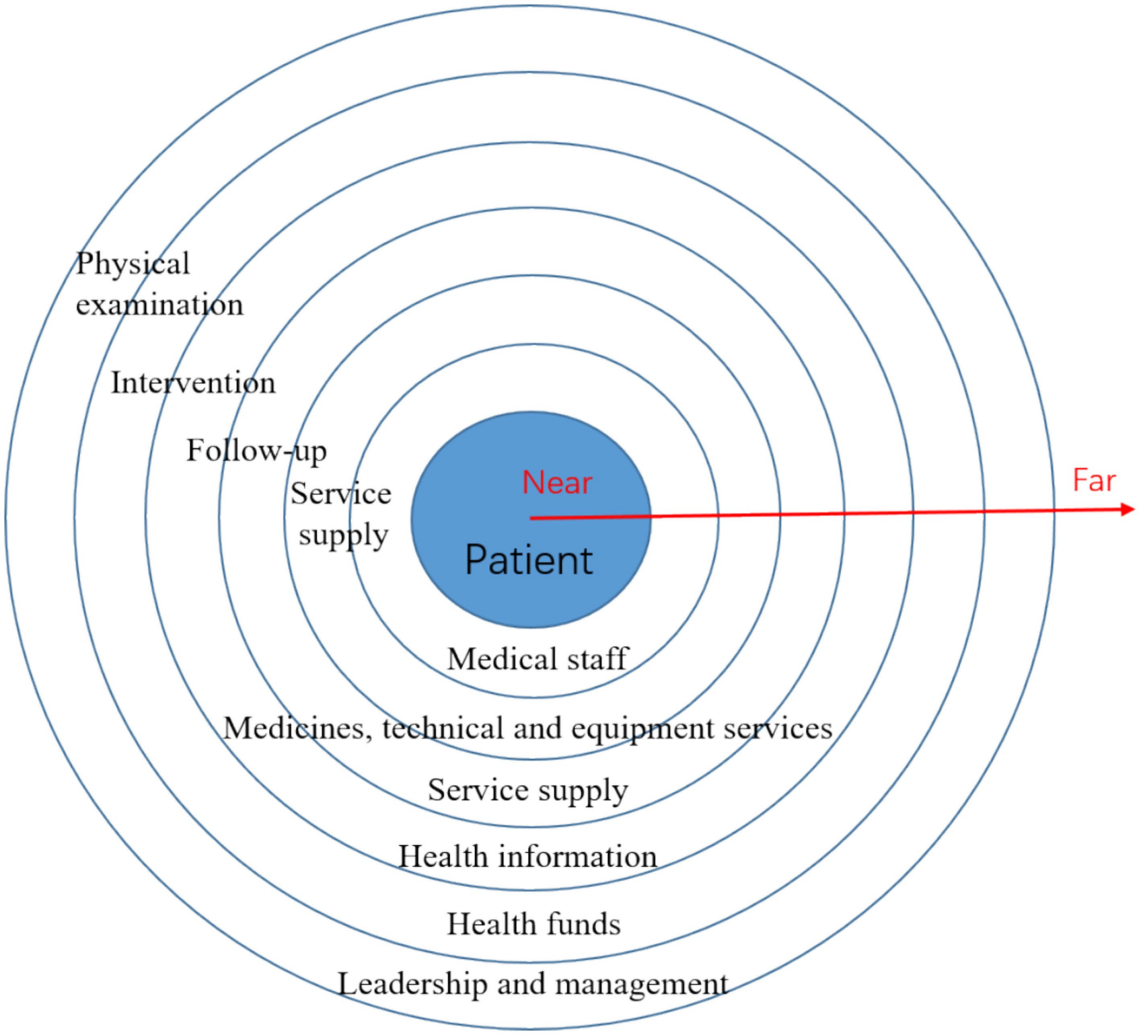
Schematic diagram of stakeholder analysis.

Figure 5 illustrates the stakeholder map for chronic disease management within basic public health services. Patients are positioned at the center, and stakeholders are categorized based on their proximity and functional relationships with patients, which include direct service providers, indirect service providers, and support service providers. This figure aims to visualize the multi-stakeholder service ecosystem and facilitate a participatory analysis of roles, interactions, and potential coordination gaps, rather than quantifying stakeholder influence.

Chronic disease management involves multiple stakeholders, whose efforts to maximize their own interests often lead to conflicts and disputes, resulting in various challenges. For instance, there is a role overlap between health promoters and medical service providers. Village doctors, for example, fulfill both medical and health promotion functions. However, from their perspective, no clear positive correlation has been established between their compensation and the treatment of chronic disease patients. Similarly, retail pharmacies, in their pursuit of profit maximization, may influence the availability of medications for chronic disease patients. Therefore, it is essential to balance the conflicts among stakeholders and identify inherent equilibrium points.

#### 3.3.2 User personas for chronic disease patients

The user persona method is a fundamental approach in service design. This method systematically collects and analyzes data on users’ behavioral characteristics, psychological needs, value orientations, and social attributes to construct representative user archetypes (39). By developing these personas, designers can gain a deeper understanding of users’ motivations, expectations, and contextual behaviors. The user persona method facilitates the identification of diverse user needs and provides qualitative and quantitative support for optimizing service processes, interaction design, and resource allocation. Centering design decisions around user persona s enables service systems to deliver more personalized, empathetic, and value-driven experiences, thereby enhancing user satisfaction and overall service effectiveness.

In this study, we employed the user persona method to construct personified user types through comprehensive user research. This approach effectively represents patient groups that share common interests and characteristics (39). Its purpose is to reflect the behavioral patterns and mental models of typical patients in pilot studies, thereby providing deeper insights into their motivations, goals, and needs. Based on service-design logic, three patient typologies were developed as empirically grounded service-design personas to represent distinct patterns of chronic disease experience and management behavior. Qualitative transcripts from semi-structured interviews (*n* = 10) and FGDs (*n* = 19) were analyzed using thematic analysis. Two researchers independently conducted open coding to capture behavioral, cognitive, and affective expressions related to medication use, lifestyle regulation, service utilization, and trust in primary care. Through axial coding, conceptually related codes were clustered into higher-order themes aligned with six recurrent domains: information acceptance (accessibility), health awareness (consciousness), family support (support), behavior modifiability (transitivity), health payment ability (payment), and medication adherence (compliance). Finally, selective coding integrated coherent bundles of themes into three interpretable personas: conservative–passive, bystander, and hearsay types.

**Figure 6:**
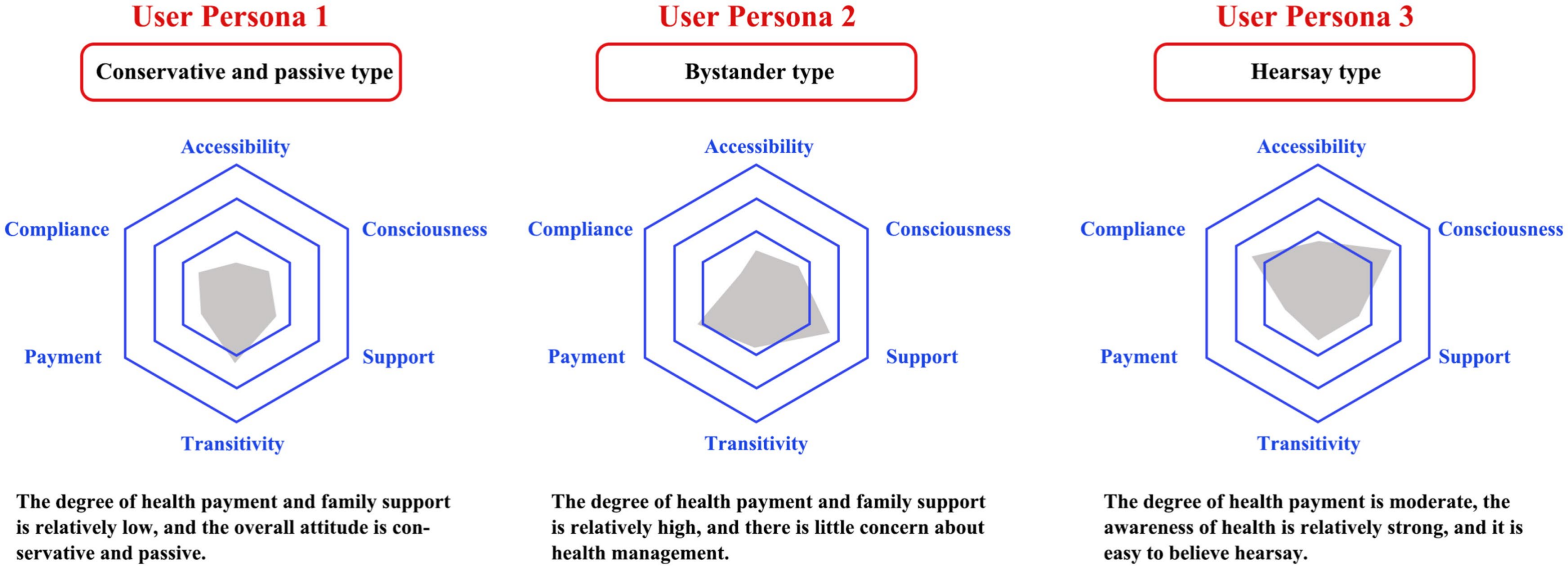
Illustrative user personas for chronic disease management derived from qualitative analysis.

Figure 6 presents three illustrative user personas—conservative–passive, bystander, and hearsay—developed from a thematic analysis of semi-structured interviews and focus group discussions (FDPs). These personas synthesize recurring behavioral patterns, perceptions, and contextual factors related to chronic disease management. They are intended as internal service design tools to support interpretation and planning, and do not represent statistically generalizable population segments. The utilization of personas and journey mapping as analytical tools aligns with recent methodological guidance in healthcare service design and patient experience research (30, 40, 41).

The three illustrative user personas are analyzed as follows: (1) Conservative and Passive Type. Behavior: The patient exhibits irregular medication adherence, typically taking medications only when they remember. They have a history of smoking and alcohol consumption, which they find challenging to quit. The patient expresses concerns regarding the side effects of medications and occasionally seeks drugs through alternative channels. They predominantly opt for community hospitals for treatment due to higher reimbursement rates and lower medication costs. Psychology: During each medication pickup, the patient listens to the doctor’s instructions but struggles to retain the extensive information provided. They show a lack of interest in health lectures and are unwilling to expand their knowledge on related health topics. While they are generally aware that complications could result in higher medical expenses, they find it difficult to alter their lifestyle habits. Holding traditional beliefs, some patient considers themselves to have lived a long life at over 70 years old and adopts a fatalistic attitude. (2) Bystander Type. Behavior: The patient demonstrates little concern for healthy eating habits, favoring preserved fish and cured meats. They participate in annual company-organized physical checkups but incur significant expenses due to chronic diseases, occasionally opting for imported medications as recommended by their doctors. Psychology: The patient expresses distrust toward community hospitals, believing that larger hospitals offer more reliable care. They exhibit indifference toward healthcare venues and avoid discussing personal health conditions, refraining from communication about them even with friends during card games. There is a noticeable lack of interest in new information, and they show no willingness to learn about health-related topics. (3) Hearsay Type. Behavior: The individual frequently visits community hospitals and proactively prepares a medicine box in advance for prescriptions. They enjoy gathering hearsay and attend free health check-ups upon hearing about them. Additionally, there is a notable interest in traditional Chinese medicine and health maintenance, often demonstrated by watching related television programs; however, the individual exhibits poor dietary habits. Psychology: There is a persistent worry about contracting serious illnesses and becoming a burden to the family. The individual is willing to try medications recommended by neighbors and considers purchasing herbal medicines advertised on television, though they feel hesitant to ask their son for financial assistance. Medication compliance is deemed acceptable.

The six domains underpinning the three patient personas were systematically synthesized through thematic coding. The key empirically coded patterns are summarized below.

Accessibility: Six participants articulated their perceptions of medication-related risks, highlighting concerns about adverse effects that, in some cases, contributed to delayed initiation or reliance on unofficial acquisition channels. Five interviewees described difficulties in retaining detailed clinical instructions delivered during brief consultations and exhibited limited engagement with health education activities. Instead, they frequently depended on informal information networks and primarily participated in no-cost or convenience-based health check-ups when available.

Consciousness: A recurrent theme across interviews was the low prioritization of dietary control. Notably, 40% of respondents acknowledged high-salt dietary habits, reflecting insufficient awareness of lifestyle-related risks. Preventive behaviors were often framed as routine compliance with workplace-organized annual physical examinations rather than proactive self-management. Three patients also expressed a willingness to trial medications based on recommendations from neighbors or acquaintances, indicating a reliance on informal health information.

Support: The family context emerged as a salient determinant of health-related behaviors. Three participants reported family histories of chronic conditions and frequently exchanged treatment experiences within their kinship networks. Household dietary environments and long-standing taste preferences were described as constraints on adherence to low-salt diets. Additionally, illness-related anxiety and fear of hospitals were voiced by four patients, often accompanied by concerns about becoming a burden on family members in the event of severe disease.

Transitivity: Entrenched lifestyle risk behaviors emerged as a consistent code cluster. In in-depth interviews, three out of ten participants explicitly reported routine alcohol consumption. Two participants characterized these habits as challenging to modify, despite acknowledging that complications could escalate long-term medical expenses. This suggests a low modifiability of behaviors and a resistance to sustained lifestyle changes.

Payment: Nine Participants expressed limited trust in community hospitals, perceiving tertiary hospitals as more clinically reliable. The utilization of community facilities was frequently described as economically motivated (e.g., higher reimbursement rates and lower medication costs) rather than driven by confidence in service quality. The financial burden associated with chronic diseases was repeatedly emphasized, with eight patients reporting the adoption of higher-cost medications when recommended by physicians, indicating a tension between affordability and perceived therapeutic value.

Compliance: Patterns of medication adherence were heterogeneous. While four patients reported stable adherence to insulin injections, forgetfulness regarding oral medication schedules was commonly observed. Forty percent of participants described intermittent dose reduction or discontinuation when they perceived an improvement in their health status, reflecting fluctuating adherence linked to subjective evaluations of risk.

#### 3.3.3 Chronic disease patient user journey map

The User Journey Map is a method that dissects the entire service process into sequential steps, typically categorized into four stages: awareness, approach, interaction, and departure (42). This framework offers a detailed and comprehensive depiction of the user experience throughout the service process, with its core components being the touchpoints encountered. From the customer’s (user’s) perspective, the User Journey Map presents a holistic view of the factors influencing the customer (user) experience across the service continuum. The primary purpose of employing this tool is to analyze the challenges faced by chronic disease patients at various stages along a timeline, thereby identifying their pain points and uncovering potential opportunities. As with personas, the journey map synthesizes patterns from 10 interviews and 19 focus group discussions (FGDs), serving as an illustrative rather than a population-representative tool. The map captures recurring experiences across four stages of care: prevention, treatment, management, and subsequent treatment.

**Figure 7:**
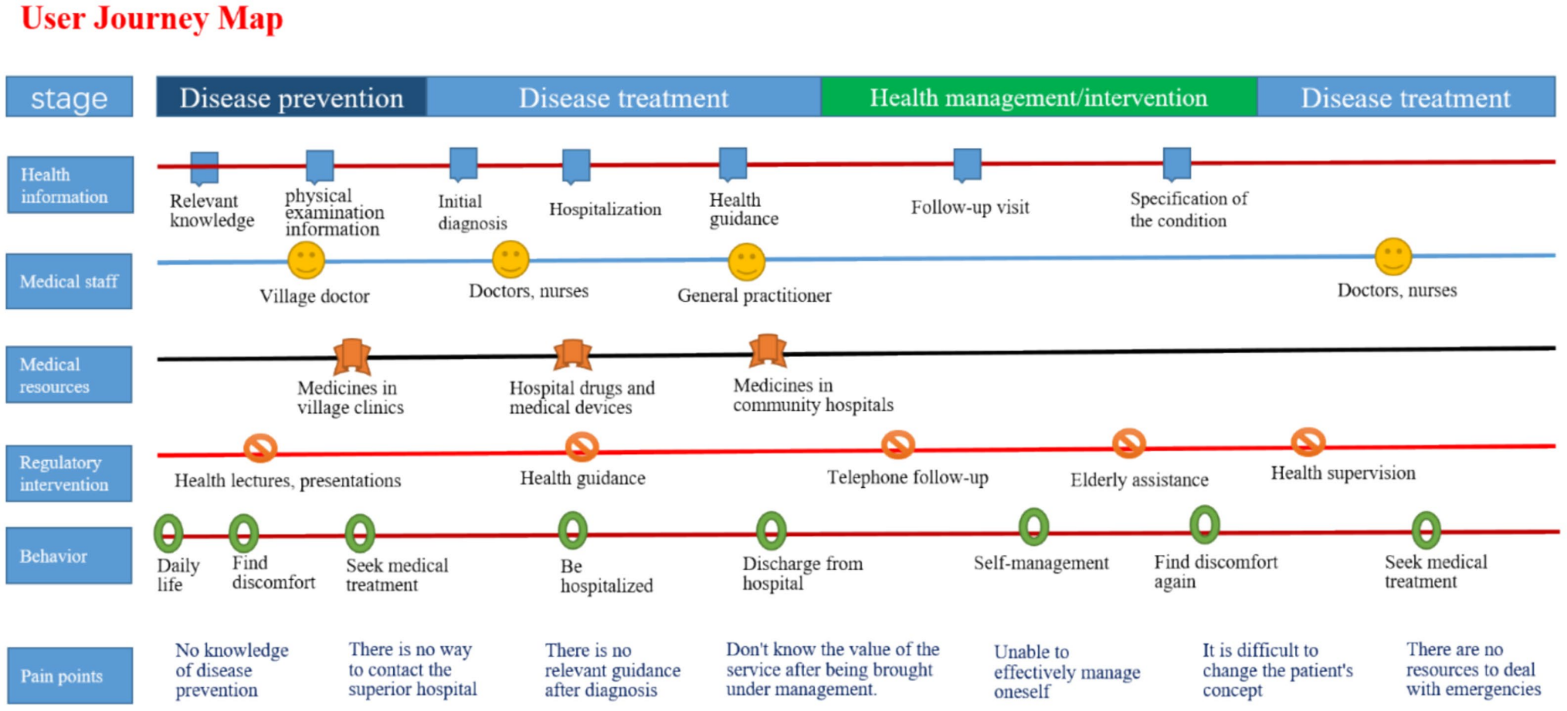
Illustrative user journey map of chronic disease care across key service stages.

Figure 7 presents an illustrative user journey map that summarizes the recurring experiences of patients with chronic diseases across four stages of care: prevention, treatment, health management, and subsequent treatment. The map integrates qualitative insights gathered from interviews and focus group discussions (FGDs) and is organized into parallel lanes that represent health information, healthcare providers, medical resources, regulatory context, patient behaviors, and reported pain points. This figure aims to highlight common experiential patterns and process-level challenges rather than to depict individual patient trajectories. The map is structured in parallel lanes that depict (from top to bottom) health information, medical staff interactions, medical resources, regulatory intervention, patient behaviors, and pain points. The key pain points identified in the user journey analysis include:

1. Lack of disease prevention knowledge. Participants described uncertainty about recognizing early symptoms or determining when medical attention was needed. Survey indicator: 30% of respondents reported difficulty assessing when discomfort warranted medical evaluation. Quote: “I wasn’t sure if my dizziness meant something serious, so I waited. (Patient, M, 63 y).”
2. Absence of direct access to higher-level hospitals. Participants reported unclear steps when transitioning from village or community facilities to higher-level hospitals. Provider insight: Two practitioners noted inconsistent referral procedures. Quote: “We used to give referral slips, but that policy changed—now patients do not know the process. (Patient, M, 73 y).”
3. Lack of continuous guidance after diagnosis. Patients expressed difficulty understanding or remembering next steps after receiving a new diagnosis. Survey indicator: 30% reported difficulty retaining treatment instructions. Quote: “After they told me I had another condition, I did not know what I should do afterward. (Patient, M, 77 y).”
4. Low perceived value of management services after enrollment. Some participants had limited understanding of the purpose of chronic disease management enrollment. Survey indicator: 40% reported they did not clearly understand standardized management content. Quote: “They said I was enrolled, but I did not know what it was for. (Patient, F, 67 y).”
5. Ineffective self-management at home. Participants described difficulty maintaining recommended home-management practices. Survey indicator: 50% reported challenges maintaining recommended diet or monitoring routines. Quote: “I forget what they told me to do once I get home. (Patient, M, 59 y).”
6. Limited behavioral change from chronic disease interventions. Participants described limited change in long-standing habits despite health education. Survey indicator: 20% reported relying primarily on informal or peer-based advice. Quote: “I still follow what my neighbors say—it feels familiar. (Patient, M, 73 y).”
7. Lack of service guidance for emergency medical situations. Participants reported difficulties deciding which medical facility to visit during urgent events. Survey indicator: 30% of surveyed patients expressed uncertainty about where to seek emergency care. Quote: “When my sugar went up suddenly, I did not know which hospital to go to. (Patient, F, 88 y).”

## 4 Discussion

The hypertension and diabetes cascades (Figures 3, 4) revealed three notable numerical discontinuities: (1) a significant proportion of diagnosed patients were not enrolled in basic public health services; (2) among those enrolled, non-standardized management practices were prevalent; and (3) the rates of treatment and clinical control were descriptively lower in groups receiving non-standardized management. These quantitative patterns were further supported by qualitative data from interviews and focus group discussions (FGDs), which highlighted uncertainties regarding follow-up pathways, variability in home self-management practices, and challenges in navigating service processes.

To enhance transparency and traceability between empirical observations and policy-oriented recommendations, we constructed a joint-display table (Table 1) that links descriptive quantitative and qualitative patterns to corresponding strategic principles, service-design-informed operational actions, and process monitoring indicators (KPIs). These indicators are intended for implementation planning and routine monitoring, rather than for evaluating outcomes or effectiveness.

**Table 1:** Joint display linking empirical observations to service-design–informed strategy principles, operational actions, and process monitoring indicators.

Empirical observation (descriptive)	Strategy principle/policy target	Operational action (service-design–informed)	Process monitoring indicator (KPI*)
Uncertainty in early symptom appraisal: 30% of surveyed patients reported difficulty determining when discomfort required medical evaluation; interviews described delayed care-seeking.	Improve process visibility for early-stage decision-making	Develop and distribute visual “When to seek care” guides; include symptom-escalation prompts in post-visit summaries at primary care facilities.	Annual monitoring: % of respondents reporting clarity on when to seek care; number of guides distributed (quarterly)
Unclear referral pathways across care levels: Patients and providers reported inconsistent referral procedures between community and higher-level facilities.	Strengthen navigation clarity across service levels	Standardize referral scripts; display district-wide referral pathway posters; reintroduce printed referral slips where feasible.	Quarterly monitoring: availability of referral posters; proportion of providers using standardized referral scripts
Limited retention of post-diagnosis instructions: 30% of surveyed patients reported difficulty remembering treatment instructions; interviews described uncertainty about next steps.	Enhance follow-up continuity and instruction retention	Provide take-home written summaries at diagnosis; deliver brief SMS reminders summarizing key follow-up steps.	Monthly monitoring: % of visits with written summaries provided; number of SMS reminders sent
Low perceived value of standardized management services: 40% of surveyed patients reported unclear understanding of standardized management content; interviews indicated low perceived relevance.	Increase transparency of chronic disease management services	Develop simple infographic sheets explaining standardized management; offer brief onboarding explanations at enrollment.	Annual monitoring: % of patients reporting understanding of standardized management; number of onboarding sessions delivered
Difficulty maintaining home self-management routines: 50% of surveyed patients reported challenges adhering to diet or monitoring recommendations.	Support clarity of home-based self-management	Provide simple actionable tools (e.g., salt-measure cards, glucometer reminder prompts); organize brief micro-education booths in community settings.	Quarterly monitoring: number of materials distributed; attendance at community micro-education activities
Reliance on informal or peer-based information: 20% of surveyed patients reported depending primarily on informal advice; interviews highlighted trust in hearsay sources.	Strengthen trusted health information channels	Disseminate verified community health messages; host monthly “ask-a-nurse/doctor” consultation tables at community health centers.	Monthly monitoring: number of messages disseminated; consultation table attendance
Uncertainty about emergency care options: 30% of surveyed patients reported uncertainty about where to seek emergency care; interviews described confusion regarding facility roles.	Improve emergency pathway comprehension	Develop emergency decision cards; display facility-role posters in community health centers and village clinics.	Annual monitoring: % of patients reporting clarity on emergency care pathways; number of cards distributed

*KPIs are proposed as process and implementation monitoring indicators only. They are not intended to assess behavioral change, clinical effectiveness, or causal impact.

### 4.1 Strategy principles: a user-centric, bottom-up approach to chronic disease management

For an extended period, healthcare services have been predominantly technology-driven, often overlooking patient needs and experiences. Similarly, in health management services, administrative priorities frequently overshadow patient-centric considerations. From a service-design perspective, the adoption of user-centric strategy principles emphasizes addressing experiential and process-related issues encountered by patients at the downstream end of the health system.

Within chronic disease management, challenges related to health information, medical personnel, pharmaceuticals and medical technology, service delivery, healthcare funding, and governance are frequently described. Given that patients remain the most directly affected stakeholders, strategy principles derived from service design prioritize improving clarity, visibility, and perceived value of services at the patient interface.

### 4.2 Operational methods: translating strategy principles into service-design actions

Guided by the strategy principles outlined above and the joint-display structure presented in Table 1, operational methods are articulated across three interrelated layers: the patient layer, the bridge (service-process) layer, and the resource layer. At each layer, operational actions informed by service design are derived from descriptive empirical observations identified through administrative data, surveys, interviews, and focus group discussions (FGDs). These actions are implementation-oriented and are accompanied by process monitoring indicators rather than outcome measures.

#### 4.2.1 Patient layer (users)

At the patient level, qualitative findings revealed variability in disease knowledge, confidence in primary care, and reliance on informal information sources. The personas derived from these patterns functioned as illustrative planning tools rather than representative population segments. Operational actions informed by these observations include the provision of simplified health education materials, the use of structured communication scripts to facilitate symptom interpretation, and the delivery of written or SMS-based summaries following a diagnosis. These actions align with the strategic principles presented in Table 1 and are associated with process monitoring indicators, such as the frequency of material distribution and patient-reported clarity, without implying behavioral or clinical effectiveness.

#### 4.2.2 Bridge layer (service process)

The bridge layer highlights recurring service-process issues identified in interviews, such as ambiguous referral steps, limited service touchpoints, and fragmented information flow. Operational actions at this layer aim to enhance process visibility and ensure consistent communication across various levels of care. Examples of these actions include standardizing referral explanations, expanding patient-facing touchpoints through written summaries or reminder tools, and restructuring the information flow to provide both providers and patients with transparent access to follow-up requirements. Table 1 summarizes how these actions are linked to process monitoring indicators, including the availability of referral materials and the completion of documentation steps.

#### 4.2.3 Resource layer (service provision)

**Figure 8:**
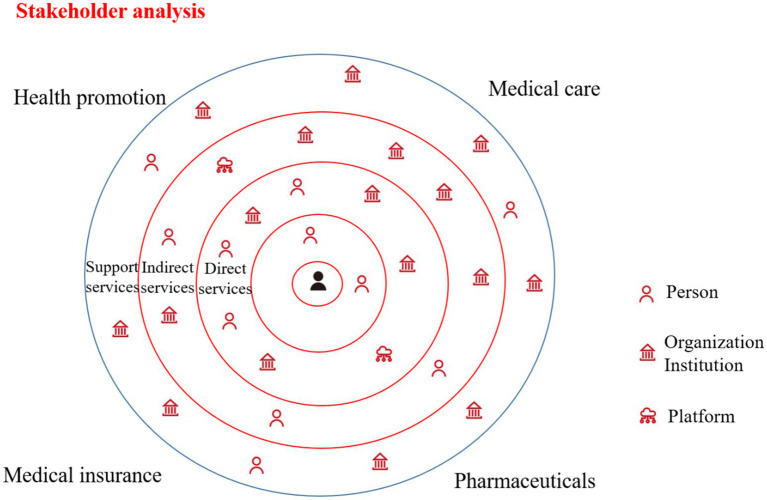
Hierarchical classification of service provision resources by proximity to patients.

Figure 8 illustrates a hierarchical organization of service provision resources in chronic disease management, arranged according to their proximity to patients. Resources closest to patients, such as frontline medical staff and essential medications, are prioritized, followed by service delivery mechanisms, information systems, funding structures, and governance arrangements. This figure supports the operational logic of prioritizing patient-proximal resources when planning service-design-informed interventions under resource constraints. Interview and focus group discussion (FGD) participants described constraints related to staffing, medication availability, equipment, digital systems, and coordination routines. These descriptive accounts informed operational recommendations that prioritize: consistency in frontline staffing; reliability of essential medications and equipment; structured service routines; improved information systems; and planning mechanisms for funding and management. It is important to note that these recommendations do not imply that changes will necessarily lead to improved control or behavioral outcomes; rather, they aim to address observable constraints that affect service consistency and visibility.

### 4.3 Service touchpoints

In this framework, service touchpoints are conceptualized as concrete expressions of operational actions at the patient interface, rather than as a distinct analytical layer. These touchpoints are essential for facilitating patients’ understanding of their care journey. Findings indicate that the existing touchpoints—primarily verbal guidance and prescription notes—may be inadequate for reinforcing self-management. Redesigning operational touchpoints may involve the incorporation of reminders, simplified written materials, or visual process aids. Such modifications aim to enhance comprehension and participation, as supported by interview data, without making causal claims regarding adherence or outcomes.

### 4.4 Governance and implementation of service design insights

Translating service design insights into practice requires governance arrangements that support the implementation and routine monitoring of operational actions identified through empirical analysis. At the front-line level, community physicians and nurses may apply persona-informed insights to tailor follow-up communication, while meso- and macro-level actors may use journey-map evidence to refine workflows and coordination mechanisms, as reflected in the monitoring indicators outlined in Table 1.

### 4.5 Limitations

This study has several limitations that must be acknowledged when interpreting the findings. First, while the administrative database provides comprehensive coverage of diagnosed hypertension and diabetes cases in Xiling District, medication adherence and lifestyle adherence were not directly measured. Treatment and control indicators were derived from recorded prescriptions and clinical measurements rather than objective adherence metrics (e.g., pill counts, pharmacy refill data, or digital monitoring). Consequently, the observed gaps along the care cascade cannot be attributed to patient adherence behaviors. Therefore, the proposed service-design actions should be interpreted as targeting process visibility and service organization rather than individual compliance. Second, the user personas and journey maps presented in this study are illustrative analytical tools rather than population-representative classifications. Personas were constructed by synthesizing recurring patterns from a limited number of interviews and focus group discussions, complemented by survey insights. Their purpose is to support interpretation and service-design thinking, not to statistically segment patients or predict behavioral outcomes. Accordingly, these artifacts should be understood as heuristic devices to guide policy discussions and implementation planning, rather than as definitive typologies. Third, potential misclassification of diabetes status may have occurred for a small subset of individuals identified solely through medication records. Although diagnostic codes and laboratory criteria were prioritized, some patients receiving antidiabetic medications for non-diabetic indications (e.g., polycystic ovary syndrome, obesity, or cardio-renal protection) may have been inadvertently included. To mitigate this risk, we applied exclusion rules for common non-diabetes indications; however, residual misclassification cannot be entirely ruled out and may have modestly influenced cascade estimates. Fourth, the quantitative component of this study was intentionally descriptive. The analyses focused on characterizing cascade patterns and identifying service gaps rather than estimating causal relationships or intervention effects. No multivariable modeling or confounder adjustment was conducted, and the study was not designed to assess effectiveness or behavioral change. Consequently, the findings provide a diagnostic foundation for service redesign rather than evidence of impact. Finally, the study was conducted within a single urban district, which may limit generalizability to other regions with different health system structures, resource levels, or population characteristics. Nevertheless, the analytical framework and service-design approach are intended to be transferable and adaptable across various settings.

## 5 Conclusion

This paper examines the fundamental public health issues associated with chronic disease management for hypertension and diabetes in the Xiling District of Yichang City, Hubei Province, China, using the service design model. It explores the application pathways and methodologies of service design theory in chronic disease policy formulation. First, the study analyzes the methodological differences between traditional policy design teams and service design teams in addressing chronic disease issues within the same region. It introduces, a service model theory for chronic disease management, which comprises three core elements: users, service processes, and service provision. This model facilitates the unraveling of complex service problems, thereby enabling a more comprehensive and precise identification of issues and exploration of the essence of services. Second, the paper proposes an approach to chronic disease policy design based on service design theory and methodologies. It identifies three tiers of issues in chronic disease management: patients, management processes, and resource provision. By departing from conventional frameworks of chronic disease policy research, this approach systematically derives a patient-centered, multi-stakeholder collaborative, and bottom-up strategy for addressing chronic disease challenges. Through service design model analysis, the proposed solutions for chronic disease management are categorized into three components: strategy, methods, and touchpoints. The methods are further divided into patient-level, bridge-level, and resource-level interventions. This paper presents a chronic disease policy design method grounded in service design theory and methodologies, enabling policymakers to optimize patient experience and perception while working within limited resources.

Although this study was conducted in Xiling District, its methodological and analytical framework possesses broader relevance for both national and international contexts. The findings may contribute to the implementation of Healthy China 2030 by providing a structured, user-centered approach to enhance the accessibility and equity of chronic disease services. The emphasis on co-creation and evidence-informed iteration aligns with the National Basic Public Health Service Standards and emerging digital health strategies. The framework resonates with the WHO People-Centered Care model and the OECD Innovation in Public Services guidelines, which advocate for participatory, cross-sectoral approaches to health governance. By emphasizing user experience as a foundation for policy adaptation, the proposed model can be applied across diverse healthcare systems—particularly those undergoing primary care reform or digital transformation. Future research should evaluate the framework’s adaptability through comparative pilot studies across regions and health systems to validate its scalability, contextual flexibility, and long-term policy impact.

## Data Availability

The original contributions presented in the study are included in the article/Supplementary material, further inquiries can be directed to the corresponding author/s.
