# Supplementary material for: Chronic disease management: policy design based on service design methods
Source: Front Public Health. 2026 Jan 13;13:1704995. doi: 10.3389/fpubh.2025.1704995 (PMC12835359; doi:10.3389/fpubh.2025.1704995)
Supplement: Supplementary file 1 [file Data_Sheet_1.PDF]

Supplementary Materials

1. A e-identified participant table
2. Interview guide
3. FGD guide
4. Coding framework/Exemplar quotes table
5. Theme map
6. A brief saturation memo

1. A e-identified participant table

Table S1. Characteristics of the 10 Interview Participants

Variable	P1	P2	P3	P4	P5	P6	P7	P8	P9	P10
**Age (years)**	88	38	73	73	63	80	83	77	67	59
**Sex**	F	F	M	M	M	F	F	M	F	M
**Diagnosis**	DM	HTN + DM	HTN	HTN + DM	HTN + DM	DM	HTN	HTN + DM	HTN + DM	HTN
**Years since diagnosis**	18 yrs	HTN 2 yrs; DM 1 yr	7 yrs	HTN 10 yrs; DM 20 yrs	HTN 6 yrs; DM 1 yr	12 yrs	5 yrs	HTN 15 yrs; DM 15 yrs	9 yrs	11 yrs
**FPG / HbA1c category**	FPG 8 mmol/L	FPG >10 mmol/L	—	HbA1c 7–7.5%	FPG >7 mmol/L	FPG 6.5 mmol/L	—	HbA1c >9%	FPG >7 mmol/L	—
**Treatment regimen**	Insulin	OHA + ACEI/ARB	ACEI/ARB	Insulin + two antihypertensive drugs	Insulin + OHA + two antihypertensive drugs	OHA	Two antihypertensive drugs	Insulin + OHA + two antihypertensive drugs	ACEI/ARB	Two antihypertensive drugs
**Standardized management**	No	Yes	No	No	No	Yes	Yes	No	No	No
**Comorbidities**	Gastric disease	Thyroid cancer	None	Gastric disease; retinopathy	None	None	None	Not reported	Fatty liver	Not reported
**Primary care setting**	Community CHC	Community CHC	Village clinic	Village clinic	Village clinic	Community CHC	Village clinic	Community CHC	Village clinic	Village clinic
**Hospitalization (past 12 months)**	No	Yes	Yes	No	No	No	Yes	No	No	No

Notes

HTN = hypertension; DM = diabetes mellitus

FPG = fasting plasma glucose; HbA1c = glycated hemoglobin

OHA = oral hypoglycemic agents

ACEI/ARB = angiotensin-converting enzyme inhibitors / angiotensin receptor blockers

CHC = community health center

"—" indicates data not available or not applicable

This table summarizes the age, sex, diagnoses, duration since diagnosis, glycemic indicators, treatment regimens, standardized management status, comorbidities, primary care settings, and recent hospitalization history of the ten interview participants included in the qualitative component of the study. These characteristics are reported to provide contextual background for the development of illustrative user personas and journey maps, and they are not intended to represent the broader

population of patients with hypertension or diabetes.

2. Interview Guide (Abbreviated Version)

Purpose

To explore patients' experiences with chronic disease diagnosis, treatment, self-management, and healthcare services, and to inform service-design–oriented analysis.

Introduction and Consent

Briefly explain the study purpose and interview process. Emphasize voluntary participation and confidentiality. Obtain verbal informed consent before starting.

Section 1. Participant Background

Age, sex, education level; living arrangement and household context; main source of income; type of chronic disease(s) and time since diagnosis; health insurance coverage.

Section 2. Diagnosis and Care Pathway

How and when the condition was first detected; where the diagnosis was made; current treatment regimen and any changes over time; follow-up experiences; medication use and challenges.

Section 3. Self-management and Disease Perception

Self-rated health status; lifestyle behaviors; monitoring practices; actions taken when results are abnormal; emotional responses and concerns related to the disease.

Section 4. Health Services Experience

Usual source of care and reasons for choosing it; experiences with community health services and family doctor programs; perceived accessibility, continuity, and usefulness of services; financial burden and insurance reimbursement experience.

Section 5. Information Sources and Support

Main sources of health-related information; role of family members or peers in disease management; participation in health education or community activities.

Section 6. Needs, Expectations, and Suggestions

Unmet needs in chronic disease management; suggestions for improving services or support; willingness to participate in education programs or peer-support activities; confidence in managing the condition and future expectations.

Closing Question

Is there anything else you would like to share about your experience with chronic disease management?

Notes

Interviews were semi-structured, and probes were used as needed to clarify responses or explore emerging themes. The guide was used flexibly to allow participants to elaborate on issues most relevant to their experiences.

3. FGD guide (Abbreviated Version)

Purpose

To explore perspectives on primary health care and chronic disease prevention policies and services from the viewpoints of policy makers, healthcare providers, and residents with chronic diseases, and to inform service-design–oriented analysis.

Introduction and Ground Rules

The moderator explains the study purpose, emphasizes voluntary participation and confidentiality, and obtains verbal consent. Participants are encouraged to share experiences openly and respectfully.

Key Discussion Domains (All Groups)

• Perceptions of chronic disease prevention as a local health priority
• Experiences with primary health care and related services
• Strengths and limitations of current policies or services
• Coordination across institutions and sectors
• Unmet needs and priority areas for improvement

Stakeholder-Specific Focus

Policy makers: policy evolution, implementation, coverage, sustainability, and governance
Healthcare providers: service delivery, workforce, information systems, financing, and care models
Residents: care experiences, accessibility, affordability, trust, and self-management support

Integrated Health Services Perspective

Discussion probes aligned with five key strategies of integrated health services: community empowerment, governance and quality improvement, transformation of care models, cross-sectoral collaboration, and creation of supportive environments.

Closing

Participants are invited to summarize key concerns and suggest feasible improvements for chronic disease prevention and primary health care services.

Notes

FGDs were semi-structured and moderated flexibly. Probing questions were used as needed. The guide is descriptive and exploratory and does not imply causal inference.

4. Coding framework/Exemplar quotes table

Theme	Category	Code	Examples of Data Excerpts
Medical service issues	Service accessibility	Shortage of doctors	"The health center is not far away, but the doctors are often not there."
		Long waiting time	"I often queue up at 8 am, but I can only see the doctor after 10 am."
		Regular follow-up visits are missing	"After they told me I had another condition, I didn't know what I should do afterward."
		Project inspection is vague	"I hope the medicine can be cheaper, and sometimes the examination items can be clearer."
		Lack of medical personnel	"There is only one chronic disease management doctor in our health center, and he has to manage hundreds of patients with hypertension and diabetes in the entire area, which is simply too much for him to handle."
		The attending doctor is not fixed	"Now every time I go, I meet a different doctor, and each doctor says something slightly different."
		No on-site follow-up service	"The doctor said they were short-staffed and couldn't make house visits. If there were any issues, they could call."
	Information accessibility	The lecture is difficult to understand	"It's too technical, and I can't understand many of the terms."
		Medical terminology is abundant	"Last year, the health center held a diabetes lecture, and I went to listen, but it was all about medical terms, such as "insulin resistance" and "glycated hemoglobin", which sounded like gibberish to me."
Individual management issues	Self-management	Physical examination becomes a mere formality	"I didn't undergo regular physical examinations before, and would only visit the clinic occasionally when feeling unwell."
		Low medication adherence	"For a period of time, I felt that my blood sugar was quite stable, so I tried to eat half a tablet less."
		Participate in fewer lectures	"I forget what they told me to do once I get home."
		Self-monitoring not comprehensive	"I don't have a blood pressure monitor at home, so I always go to the health center to have my blood pressure measured."
	Family environment	Family chronic medical history	"Both my father and a younger brother have a history of hypertension."
		Difficulty in diet management	"I know that too much salt is bad for blood pressure, but I've been used to it for decades and can't really change it in a short time."
	Economic burden	Low reimbursement rate	"But going to the county hospital costs more and the reimbursement ratio is lower."
		Self-testing supplies are expensive	"Regarding blood sugar, my son bought me a blood glucose meter last year, which cost over 300 yuan. The test strips are quite expensive; a box of 50 strips costs over 80 yuan."
		Heavy burden of medication costs	"Some pharmacies are quite expensive."
	Blindly following non-standard treatment	The gap of ineffective folk remedies	"Sometimes when I hear from neighbors that a certain traditional Chinese medicine or dietary therapy is effective, I will give it a try, but most of the time, the results are not noticeable. In the end, I still have to come back to taking western medicine."
		Blindly following	"I still follow what my neighbors say—it feels

		recommendations from fellow patients	familiar.”
Experience of medical treatment management	Self-management anxiety	Lack of initiative in physical examination	“They said I was enrolled, but I didn’t know what it was for.”
		Self-medication with luck	“My daughter says I'm "not willing to cry until the coffin is seen", and I know it's not right, but when you get old, you always have a bit of a fluke mentality.”
		Diet matching confusion	“I don't know how to balance my diet. Sometimes, if I eat too little, I worry about malnutrition, but if I eat too much, I worry about high blood sugar.”
	Hospital selection dilemma	Insufficient confidence in basic medical care	“For minor illnesses, I prefer to see a doctor at the health center, but for serious illnesses, I still want to go to the county hospital.”
		Trust and reliance on equipment in large hospitals	“But if I really have a serious illness, I still want to go to a big hospital. I feel more at ease there because the doctors are highly skilled and the equipment is good.”
		Recognition of the convenience of community hospitals	“Mainly in community hospitals, and only go to large hospitals when there are problems.”
	Expectations for medical services	Health guidance needs	“When my sugar went up suddenly, I didn’t know which hospital to go to.”
		On-site service is eagerly desired	“I hope the doctor can tell us more about how to eat and exercise. It would be great if someone could come to visit us older people.”
		Organize health lectures	“I wasn’t sure if my dizziness meant something serious, so I waited.”
		Reduce the cost of medicinal materials	“I hope the medicine can be cheaper.”
		Improve primary healthcare	“We used to give referral slips, but that policy changed—now patients don’t know the process.”
		Increase the proportion of medical insurance reimbursement	“I hope the reimbursement ratio can be increased, especially the reimbursement limit for outpatient medication.”

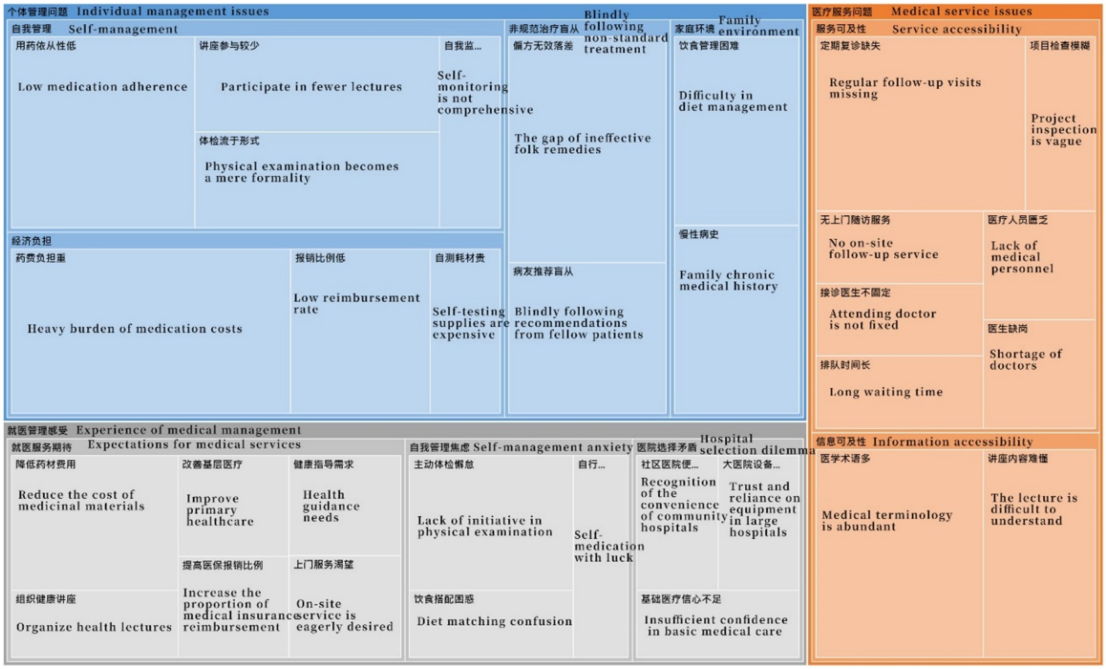

5. Theme map

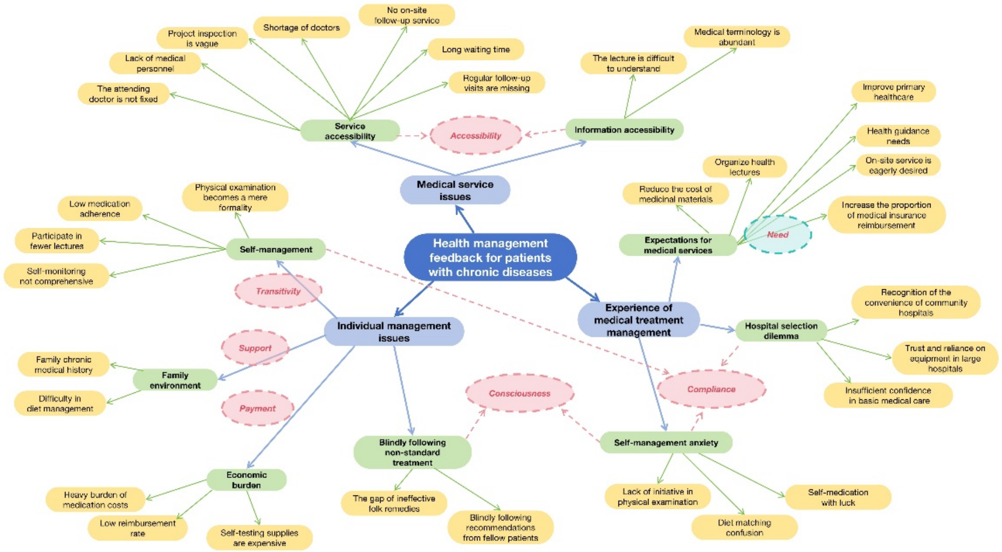

6. A brief saturation memo

To ensure methodological rigor, this study adopted thematic saturation as the criterion for terminating data collection. Following the completion of the first six in-depth interviews, the research team conducted open coding to identify a preliminary set of core themes. Subsequently, a rolling analysis strategy was implemented: for every additional two interviews collected and analyzed, the new data were systematically compared with the existing thematic framework to ascertain whether new conceptual categories emerged or if substantial revisions to existing themes were necessary.

The analysis revealed that starting from the 11th interview, the information provided by participants became repetitive, with no new themes emerging. To ensure caution and confirm stability, two additional interviews were collected and analyzed, resulting in a total of 15 interviews. Through team discussions, it was confirmed that the final five interviews were adequately captured by the established thematic structure and did not yield new theoretical insights. Based on this, the study concluded that data saturation had been achieved and that the theoretical framework had reached a stable state.
